# Rethinking success in public health education and promotion: a narrative ownership framework for sustainable health behavior change

**DOI:** 10.3389/fpubh.2026.1852833

**Published:** 2026-09-28

**Authors:** Tsuyoshi Okuhara, Hiroko Okada, Emi Furukawa

**Affiliations:** 1 Department of Health Communication, School of Public Health, Graduate School of Medicine, The University of Tokyo, Tokyo, Japan; 2 University Hospital Medical Information Network (UMIN) Center, The University of Tokyo Hospital, Japan

**Keywords:** health behavior change, hypothesis and theory, narrative ownership, person-centered care, program evaluation, public health education and promotion

## Abstract

Public health education and promotion has largely evaluated success in terms of measurable behavioral outcomes. While this outcome-centered logic has advanced intervention science, behavior change is frequently not sustained once programs end, and outcome indicators alone cannot show how change is experienced, interpreted, and held over time. The objective of this paper is to advance and operationalize the hypothesis that outcome-only definitions of success are epistemologically incomplete and ethically fragile. We proceed conceptually, identifying two structural limitations of outcome-centered evaluation, the invisibilization of temporal experience and the externalization of agency through optimization logic, and we then define a complementary evaluative dimension, specify its measurement model, and derive testable predictions. Narrative ownership is defined as the degree to which behavior change experiences, including lapses, are framed as part of an ongoing process, remain repairable through reinterpretation, and are held with a sense of authorship. We distinguish this construct from self-efficacy, autonomous motivation, self-concordance, identity-based motivation, psychological flexibility, self-compassion, and narrative identity, and we commit the framework to a falsifiable claim of non-redundancy. We propose a two-axis model crossing behavioral outcomes with narrative ownership, yielding four illustrative quadrants: integrated success, brittle success, meaningful preparation, and disempowered stagnation. We specify a second-order formative measurement model, three hypotheses in operational form, decision rules for combining the two criteria in program evaluation, and the conditions under which the framework should be abandoned. We also distinguish low narrative ownership arising from constrained options from low narrative ownership arising from interpretive processes, so that the framework does not individualize structural disadvantage. We conclude that the Narrative Ownership Framework offers a testable model for rethinking success in health behavior change, and that it may help public health educators, clinicians, and digital intervention designers distinguish short-term behavioral improvement from more durable, person-centered change.

## 1 Introduction

In health education and promotion, program evaluation has predominantly defined success in terms of measurable behavioral outcomes. While this outcome-centered logic has strengthened intervention science, it can overlook how change is experienced, interpreted, and sustained in the context of everyday life. This paper argues that such an approach is epistemologically incomplete and ethically fragile, and proposes the concept of “narrative ownership” as a complementary evaluative dimension.

The standardization of behavior change techniques (BCTs) (1), the proliferation of nudge-based interventions (2), and the expansion of digital behavior change interventions (3) have repeatedly demonstrated intervention effectiveness. However, meta-analyses indicate that effect sizes remain modest and vary across contexts (4), raising questions about the intervention design and evaluative frameworks that define success.

The gap between producing change and sustaining it is well documented across behavioral domains. In smoking cessation, most quit attempts end within the first days, and a substantial proportion of those who achieve initial abstinence return to smoking over the following year (5). Behavioral weight management programs typically produce meaningful reductions by the end of treatment, which are then followed by gradual regain (6), and this regain is accompanied by deterioration in psychological outcomes as well as in weight (7). In digital behavior change interventions, declining use is a structural feature of engagement rather than an anomaly, and participation often falls steeply within weeks of enrollment (8). Reviews of theories of maintenance have concluded that the processes that sustain behavior differ from those that initiate it, and that maintenance itself remains comparatively under-theorized (9).

These patterns are ordinarily interpreted as evidence that interventions need to be more powerful, better targeted, or better timed. However, they also admit of a different reading. If the success of a program is certified on the basis of a difference between two measurements, and if that certification is complete once the difference has been observed, then whatever governs what happens after the second measurement falls outside the definition of success rather than outside the intervention itself. The problem of maintenance is thus partly a problem of evaluative criteria, and not only a problem of mechanisms.

Behavioral outcomes can record changes, but they do not capture how individuals experience and interpret the process of change, including conflicts and lapses (10, 11). As behavior changes unfold over time (12), the ability to re-narrate setbacks substantially influences the sustainability of change (13, 14). Moreover, outcome-centered evaluation stands in tension with the principles of person-centered care and shared decision-making, which conceive of health behavior change as a process in which individuals make sense of and take ownership of changes within their values and life histories (15, 16). If outcome attainment alone defines success, agency falls outside of evaluation and the alienation or dependence behind numerical improvement remains invisible.

To develop this argument, we present the Narrative Ownership Framework (NOF) as a hypothesis-generating conceptual model for health behavior change. The NOF redefines success along two dimensions, behavioral outcomes and narrative ownership. Narrative ownership refers to a state in which lapses and interruptions are framed as part of an ongoing process, remain repairable through reinterpretation, and are held with a sense of authorship. This paper makes three contributions: it identifies the epistemological and ethical limitations of outcome-centered evaluation, introduces narrative ownership as a theoretically distinct construct, and derives a two-axis model and three testable hypotheses for future research.

## 2 Structural limitations of evaluation in behavior change research

Across behavior change techniques, nudges, and digital interventions, evaluation has largely treated measurable improvement as the primary indicator of success (1–4). We argue that this evaluative logic has two structural limitations that justify the introduction of a new theoretical construct: one epistemological and one ethical.

### 2.1 Static outcomes: the invisibilization of temporal experience (epistemological limitation)

In the prevailing evaluation frameworks, success in behavior change is operationalized as the frequency of target behaviors or numerical indicators (1, 17). Indicators, such as step counts, medication adherence rates, and HbA1c levels, ensure measurability and objectivity. However, the dynamic experience of achieving these outcomes has not been theoretically addressed (18). In most studies, the difference between baseline and post-intervention scores, expressed as effect sizes, serves as the criterion for success (19). This point-to-point comparison structurally discards the processual quality of change that individuals experience between two timepoints: hesitation, temporary interruptions, and reinterpretation (18, 20). While the evaluation precisely measures these differences, it does not theorize the temporal structure of the change itself. This structure detaches behavior from the narrative context of a person’s life and reinforces a static understanding in which numerical improvement alone defines success (21, 22).

### 2.2 Externalized agency: the relocation of success attribution (ethical limitation)

The second limitation lies in the logic of outcome optimization, which attributes the drivers of success not to individuals’ meaning-making but to the sophistication of the intervention design. Nudges and highly personalized algorithms can guide behavior without necessarily engaging in deliberation or reflection (23). Success is often understood as a triumph of architecture, rendering an individual’s authorship invisible—their sense of having chosen and taken ownership of the change (23, 24). Existing evaluation frameworks precisely measure what has changed and to what extent, yet they pay little attention to how change is experienced by the individual or under what kind of narrative understanding it is sustained. Together, these epistemological and ethical limitations suggest that understanding the sustainability and ethical soundness of behavior change requires an evaluative dimension beyond outcome indicators.

## 3 Narrative ownership: the missing evaluative dimension

The limitations identified above highlight the need for an additional evaluative dimension, “narrative ownership.”

### 3.1 From “event” to “experience”

Behavior changes are both observable events and lived experiences. For individuals, change unfolds as a nonlinear process in which fragments resolve, conflicts emerge, lapses occur, and renewed efforts accumulate over time (25, 26). In smoking cessation, for example, whether a relapse is incorporated into a fixed narrative of “I am weak-willed” or reinterpreted as “my coping plan needs revision” can set future trajectories on divergent paths (5, 27, 28). Therefore, the same event can have different meanings, depending on how it is situated within one’s temporal self-understanding. An outcome-centered evaluation can record whether a lapse occurs but not how the experience is framed, repaired, or owned within the individual’s narrative.

### 3.2 Definition and theoretical positioning of narrative ownership

To address this gap, this study proposes the concept of narrative ownership. Narrative ownership is defined as a state in which the experience of behavior change, including lapses and interruptions, is framed as part of an ongoing process (process framing), remains repairable through reinterpretation within one’s personal narrative (narrative repair), and is held with a sense of authorship as one’s own story (authorship). The three constituent elements are as follows. (1) Process framing: Situating individual lapses and setbacks within a continuous process that includes their surrounding context, what triggered them, and what was learned from them. When this framing is in place, individual lapses become comprehensible, not as isolated events, but as moments within an ongoing process. (2) Narrative repair: Individual lapse episodes are not fixed as irreversible failures, but are reinterpreted within one’s personal narrative and reconnected to renewed effort. (3) Authorship: change is attributed not as an externally imposed result but as “one’s own story,” one that can be told in one’s own words.

Narrative ownership draws on narrative research traditions, including narrative identity, which explains how people understand themselves through stories (29); narrative coherence, which addresses temporal consistency in personal narratives (30); and the illness narrative, which examines biographical disruption and narrative reconstruction in illness (14, 31, 32). However, the NOF is not concerned with describing how the experience of behavior change is narrated per se, but how the narrative is incorporated into success evaluation; specifically the question of whether change, including lapses, is framed as a process, remains repairable, and is held with authorship.

Furthermore, the NOF is conceptually distinct from the major theories of behavior change (Table 1). Self-efficacy addresses beliefs about one’s capacity to perform behaviors (33, 34), while self-determination theory (SDT) addresses motivational autonomy (35). Existing theories explain behavioral performance and its maintenance mechanisms; however, the question of how change is experienced falls outside their scope (9). The focus of the NOF is not whether behavior has been achieved, but whether the experience of change, including lapses, is held as the individual’s own narrative.

**Table 1:** Conceptual distinction between narrative ownership and related constructs.

Construct	Core question	Primary focus and level of analysis	Treatment of lapse	Relation to narrative ownership
Self-efficacy (33, 34)	Can I do this?	Belief in capability; task-specific	A threat to efficacy beliefs	The NOF presupposes but is not reducible to efficacy beliefs; a person may retain confidence in the behavior while ceasing to hold the change as their own account
Autonomous motivation and integrated regulation (35)	Why do I do this?	Quality of the reasons for acting; congruence with the value system	A signal of incomplete internalization	High motivational quality does not guarantee that a lapse remains narratively repairable; the two are expected to be related but separable
Self-concordance (36)	Is this goal really mine?	Ownership of the goal at the point of adoption; goal level	Goal abandonment or non-attainment	Self-concordance concerns ownership of the goal before pursuit; narrative ownership concerns ownership of the unfolding experience, including its interruptions
Identity-based motivation (38)	Does this behavior fit who I am and who I am becoming?	Identity–behavior congruence and the reading of experienced difficulty; identity level	Difficulty may be read as evidence that the behavior is not for people like me	The closest of the adjacent constructs. It explains motivation through identity congruence during pursuit, whereas the NOF specifies how a completed lapse is framed, repaired, and authored, and functions as an evaluative rather than a motivational construct
Narrative identity (29, 30)	Who am I across my life?	Coherence of the life story; whole-life level	Biographical disruption	The NOF operationalizes narrative concepts at the level of a bounded behavior change episode for the purpose of evaluation
Meaning making (40)	What does this event mean for me?	Discrepancy between global and situational meaning; stressful-event level	An occasion for meaning making and adjustment	Meaning making addresses adjustment to adverse events in general, whereas the NOF specifies three components tied to behavior change lapses and to program evaluation
Psychological flexibility (37)	Can I act on my values while experiencing discomfort?	Trans-situational regulatory capacity; dispositional	An internal experience to be accommodated without avoidance	Psychological flexibility is a general capacity, whereas narrative ownership is a state-level, content-specific property of a particular change episode
Self-compassion after lapse (39, 42, 43)	How do I treat myself when I fail?	Affective and attitudinal stance toward the self; episode level	An occasion for self-kindness rather than self-criticism	Related and expected to correlate moderately with narrative repair, but distinct: a compassionate affective stance does not by itself constitute narrative integration of the lapse into an ongoing account
Recovery identity (28)	Who am I becoming in recovery?	Group-based identity transition; social-identity level	Relapse as identity threat	Domain-specific to addiction and grounded in group membership, whereas the NOF is domain-general and located in the individual’s account of change
Narrative ownership (NOF)	Is this change, including its interruptions, still my story?	Process framing, narrative repair, and authorship; a bounded behavior change episode including lapses	Framed as part of an ongoing process, reinterpreted and repaired within one’s account, and held with authorship	—

NOF, Narrative Ownership Framework. The constructs listed above are treated as conceptually adjacent rather than mutually exclusive. The framework commits to a testable claim of non-redundancy: narrative ownership should predict post-lapse re-engagement and long-term maintenance over and above these constructs. If it does not, narrative ownership should be regarded as redundant with existing constructs (Table 2).

In particular, integrated regulation within SDT’s Organismic Integration Theory addresses motivational quality—whether the reasons for a behavior are congruent with one’s value system (35). However, a high motivational quality does not guarantee that the experience of change is held as a repairable narrative. For example, a patient may have internalized exercise as “important for my health” (i.e., integrated regulation), yet interpret a single interruption as an irreversible failure—“I knew I couldn’t keep it up.” The NOF theoretically captures this discrepancy and distinguishes it from existing behavior change theories.

### 3.3 Distinguishing narrative ownership from adjacent constructs

Narrative ownership is conceptually adjacent to several established constructs, and the contribution of the NOF depends on specifying where the boundaries lie (Table 1). These constructs differ from the NOF first in their unit of analysis: self-efficacy, autonomous motivation, self-concordance (36), psychological flexibility (37), and narrative identity are properties of persons, of goals, or of life stories, while narrative ownership is a property of a bounded episode of change—more specifically, of the stance an individual takes toward its interruptions.

They differ from the NOF also in function. The adjacent constructs are motivational or dispositional, and they are invoked in order to explain and predict behavior. By contrast, narrative ownership is proposed as an evaluative construct that supplies a second criterion against which programs and trajectories of change are judged; two constructs can be closely related to one another and still perform different roles within a theory. Moreover, the NOF commits itself to a falsifiable claim of non-redundancy: we predict that narrative ownership will account for variance in post-lapse re-engagement and long-term maintenance beyond the variance explained jointly by these constructs (Table 2). If it does not, narrative ownership should be regarded as a relabeling of what is already available, and the NOF should be abandoned.

Two constructs lie particularly close to the NOF and warrant separate comment. Identity-based motivation concerns the way in which difficulty experienced during the pursuit of a goal is read against identity (38), whereas narrative ownership concerns the way in which a lapse that has already occurred is subsequently framed, repaired, and held with authorship. Self-compassion following lapse describes an affective stance toward oneself (39); although a non-condemnatory stance and narrative repair are expected to be correlated, treating a failure kindly does not by itself integrate that failure into a continuing account of change. Meaning making, similarly, addresses the reconciliation of situational and global meaning after adverse events in general (40), whereas the NOF specifies three components tied to the lapses that occur within a deliberate attempt to change behavior.

On this basis, we argue that narrative ownership should be treated not as a variant of motivation or confidence, but as a distinct interpretive dimension of behavior change. The central theoretical claim of the NOF is that two individuals with equivalent behavioral outcomes may differ substantially in the degree to which change is sustainable, repairable after lapse, and experienced as their own. If so, evaluation frameworks that omit narrative ownership are likely to misclassify both success and failure.

### 3.4 Reinterpreting existing findings

Although the NOF is new, the phenomena that it postulates are not unobserved. Several bodies of evidence are consistent with the NOF, none of which was designed to test it, and reading them together forms part of the case for treating narrative ownership as a candidate evaluative dimension.

First, the abstinence violation effect describes how a single lapse precipitates a return to the prior pattern when it is attributed to a stable personal failing, whereas the same lapse attributed to a specific and modifiable situation does not (13, 27). What differs between these two cases is not the behavior that is recorded but the way in which that behavior is placed within the individual’s narrative, and outcome-centered evaluation registers the lapse identically in both.

Second, longitudinal research on narrative identity has shown that features of the way people narrate difficult episodes predict subsequent trajectories in mental health over periods of years, independently of the events narrated (41). The narrative properties of an account carry predictive information and are not merely of expressive interest.

Third, experimental and correlational studies of responses to dietary and other lapses suggest that a non-condemnatory stance toward oneself is associated with the resumption of the behavior and not with permissiveness (42, 43). The concern that treating lapses as repairable serves to license them is therefore not well supported, although this evidence addresses the affective dimension of the response to lapse and not its narrative dimension, and much of it is cross-sectional.

Fourth, engagement research in digital interventions has consistently found that use declines sharply and that the users who persist are not simply those who received more prompts (8, 44). Effective engagement has been argued to depend on the personal relevance that users construct for themselves (i.e., the meaning attached to use), which stands closer to the authorship component of the NOF than to exposure or dose.

None of these literatures measured narrative ownership as it is defined here, and none of them can be treated as confirmation of the NOF. Their convergence nevertheless establishes something weaker but still necessary, namely that the dimension proposed here is not merely conceivable but has appeared repeatedly, and unnamed, at the margins of existing findings.

### 3.5 Operationalizing and testing the NOF

A hypothesis and theory contribution must specify not only what it claims but also what would show the claim to be false. This subsection therefore states the measurement model that the NOF requires, sets out three hypotheses in operational form, and specifies the conditions under which the framework should be abandoned.

#### 3.5.1 Measurement model

We propose that narrative ownership be modeled as a second-order formative composite of three first-order factors, each of which is measured reflectively. The formative specification at the second order follows from the definition of the construct. The three components are not interchangeable indicators of a common cause, since an individual may frame lapses as processual while holding no sense of authorship, and the removal of any one component alters what the construct denotes and does not merely reduce its reliability. Misspecifying such a construct as a common factor can bias structural estimates severely, even where model fit is acceptable (45). Within each component, by contrast, items are conceived as manifestations of a common underlying state and are appropriately modeled reflectively.

Two consequences follow for measurement practice. The three subscales should be reported as a profile, and a single averaged total should not be treated as the construct itself, because equal weighting of formative components is an assumption and not a definition. The validity evidence required is correspondingly specific, comprising convergent, discriminant, criterion, incremental, and longitudinal evidence, with measurement invariance established before change scores are interpreted (46, 47). Table 2 sets out these requirements together with the evidence that would satisfy each of them and the evidence that would not. In structural models, narrative ownership will be represented as the higher-order formative composite estimated from the three first-order factors, whereas descriptive reporting will retain the three-subscale profile.

#### 3.5.2 Hypotheses

For the NOF to function as a hypothesis and theory contribution, its central claims must be stated in testable form. We therefore derive three hypotheses concerning prediction, intervention responsiveness, and experiential and relational consequences. All tests presuppose the development of a narrative ownership scale with process framing, narrative repair, and authorship as subscales, following established practice in the measurement of narrative constructs (48, 49), and the confirmation of discriminant validity relative to self-efficacy and autonomous motivation (Table 2).

*Hypothesis 1* (predictive validity): Even among individuals with equivalent behavioral outcomes at the end of an intervention, those with higher narrative ownership will show greater long-term maintenance and post-lapse re-engagement (41). This can be tested through a longitudinal cohort study that examines whether narrative ownership predicts maintenance trajectories at 12 months while controlling for initial improvement and self-efficacy.

*Hypothesis 2* (intervention responsiveness): If such an association is established, the next question is whether narrative ownership can be enhanced through interventions. We hypothesize that incorporating reflective support features into digital interventions would enhance medium- to long-term sustainability with narrative ownership as a mediating variable (44). This can be tested through a randomized controlled trial that manipulates reflective prompts using mediation analysis.

*Hypothesis 3* (experiential and relational consequences): We predict that among individuals who have experienced lapses, higher narrative ownership will be associated with lower self-stigma and a better-maintained therapeutic alliance, and that this association is stronger in behavioral domains marked by stigma or recurrent relapse (50). This can be tested through longitudinal studies of individuals who have experienced lapses.

This prediction has been deliberately narrowed relative to earlier formulations. Associations with reduced self-stigma and a preserved alliance would constitute evidence of psychological and relational benefit; however, they would not establish the ethical validity of the NOF. Ethical justification also requires consideration of autonomy, beneficence, justice, the distribution of power, the risk of coercion, and the adjudication of competing values. The NOF speaks to one part of this question, namely the conditions under which change can be held as one’s own, and does not settle the remainder.

Table 3 specifies each hypothesis in operational form, including design, population, exposure, measures, timing, covariates, analysis, and the result that would disconfirm it. The numerical criteria given there are proposed as worked examples that render the hypotheses testable in principle; they are not established standards, and each should be justified and pre-registered with reference to the behavior under study.

#### 3.5.3 Conditions under which the NOF should be abandoned

Taken together, the NOF should be abandoned if narrative ownership shows no incremental prediction of post-lapse re-engagement beyond adjacent constructs, if the three components prove to be empirically inseparable, or if the two evaluative axes correlate so strongly that the second conveys no additional information. These criteria are specified in Tables 2 and 3 in advance of any data; where numerical thresholds are used, their status as conventional benchmarks from the measurement literature (51, 52) or as deliberately chosen heuristics is stated explicitly.

## 4 Ethical fragility: why outcome-only success is brittle

This section analyzes the structural risks that arise when process framing, narrative repair, and authorship are excluded from success evaluations, and conceptualizes them as ethical fragility. Ethical fragility refers to the risk that, even when short-term outcomes are achieved, an individual’s capacity to interpret and sustain change as their own—and thus to exercise relational autonomy (53)—may be structurally undermined. This fragility becomes visible as three distinct layers in the absence of each of the three NOF components.

### 4.1 Loss of process framing and the reduction of learning opportunities

When the first element of the NOF, process framing, is not incorporated into the evaluation, individual lapses tend to be treated as isolated points of failure rather than as moments within a continuous process. Lapses stripped of temporal context become errors that need to be corrected immediately (54), thereby reducing opportunities for individuals to reposition the event within their own trajectory of change and learning (27). For example, if a lapse in dietary self-management triggers an algorithm that instantly recalculates a meal plan without giving the patient the opportunity to reflect on why the lapse occurred, the event is not experienced as a meaningful episode within an ongoing process. Instead, isolated errors are processed by the system.

### 4.2 Absence of repair and the emergence of self-stigma

When the second element of the NOF—narrative repair, defined as the reinterpretation and repair of lapses within one’s narrative—fails to operate, lapses are experienced not as opportunities for learning but as breakdowns in one’s story of change. This absence can harm identity. When evaluation structures implicitly assume a binary fall from success (1) to failure (0), a lapse, such as drinking during an attempt at abstinence, may be fixed not as a moment for renewed effort but as proof of personal weakness (13, 27). Evaluation frameworks that lack narrative ownership may leave little institutional space for lapses to be repaired within an individual’s narrative. We therefore hypothesize that such structures reinforce self-stigma and strain the therapeutic relationship, as specified in Hypothesis 3 (50).

### 4.3 Hollowing of authorship and the alienation of behavior

When the third element of the NOF—authorship, defined as the sense that change is one’s own—is not secured, behavior may fail to take root in an individual’s life narrative, even when outcomes are achieved. This absence results in a hollowing of authorship. When success is primarily experienced in response to environmental design or notifications, individuals may become beneficiaries of the change without becoming authors (21). As external support weakens, the sustainability of behavior may become more fragile. For example, two patients may both meet step-count targets while using the same application. For one of them, the notifications function as scaffolding around a purpose that the patient is able to state, and the walking is connected to something that the patient intends. For the other, the notifications supply the entire occasion for walking, and nothing in the practice has been connected to anything that the patient would otherwise pursue. Only in the second case is the behavior achieved without being owned, and outcome-centered evaluation cannot distinguish between the two.

Taken together, these three layers of ethical fragility represent the structural risks that arise when the components of the NOF are excluded from the success evaluation.

### 4.4 Supported authorship: scaffolding and external control

The analysis above should not be read as an argument that supported behavior is less owned. Relational accounts of autonomy hold that agency is constituted, and not diminished, by relationships and environments (53), and person-centered care rests on the same premise (15). Reliance on reminders, clinicians, family members, or assistive systems is not in itself evidence of reduced authorship.

The relevant question is whether the individual is able to give an account of the supported behavior as their own. Support that furnishes the conditions for a purpose that the individual can articulate functions as scaffolding (supported authorship), while support that substitutes for that purpose, or that operates by circumventing deliberation altogether (23), functions as external control, and external control alone hollows authorship. This distinction matters in digital contexts, where self-tracking can either extend a purpose the user already holds or install an individualized discipline in place of one (55). It is also empirical and can be assessed, since it concerns what the individual is able to say about the behavior and not how much assistance the individual receives.

## 5 Toward a two-axis evaluation model: integrating outcome and narrative

To address this ethical fragility, we propose a two-axis evaluation model that treats behavioral outcomes and narrative ownership as conceptually distinct dimensions.

### 5.1 Defining the two evaluative axes

The proposed model extends the conventional one-dimensional evaluation of success based solely on behavioral outcomes to a two-dimensional framework defined by two conceptually distinct axes (Figure 1). The first axis represents behavioral outcomes (vertical axis), referring to the degree of achievement of objectively measurable indicators, such as step counts, medication adherence rates, and clinical markers. The second axis represents narrative ownership (horizontal axis), referring to the extent to which change experiences, including lapses, are framed as part of an ongoing process (process framing), reinterpreted, and repaired within one’s narrative (narrative repair), and held with a sense of authorship (authorship). Even when behavioral outcomes are equivalent, the degree of narrative ownership may differ. Treating these axes as conceptually distinct does not imply that they are empirically unrelated. Rather, it highlights their conceptual independence; achievement in one dimension does not guarantee achievement in another.

**Figure 1:**
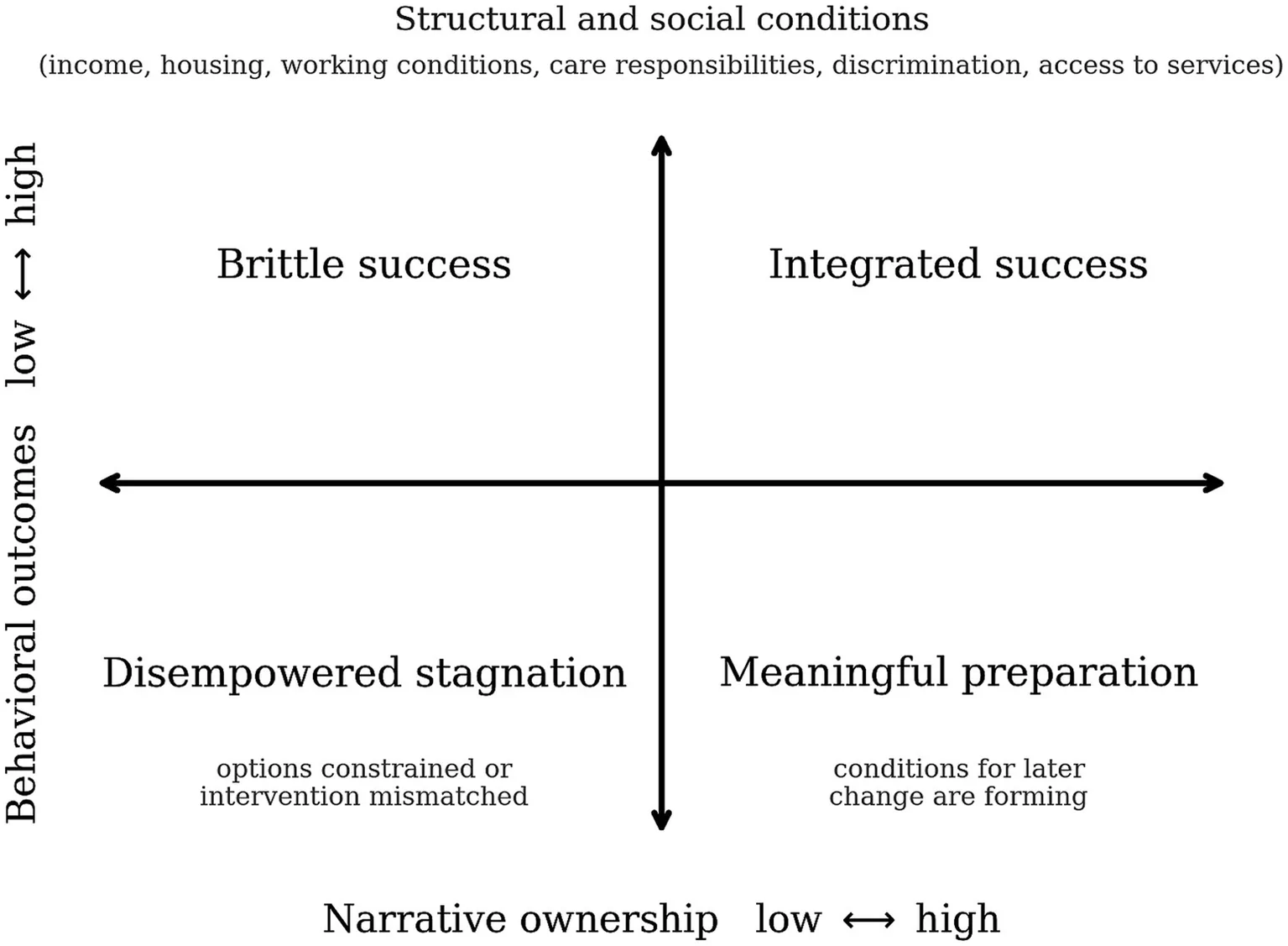
Two-axis evaluation model of behavior change: Behavioral outcomes × narrative ownership. Both axes are continuous, and the framework proposes no cutoffs; the four quadrants are illustrative conceptual states that describe configurations of person, intervention, and context, and they are not categories of persons. Both axes are situated within the structural and social conditions that determine the options available to the individual.

Whether the two axes are empirically independent is an open question that the NOF does not presume to settle. We therefore state a criterion in advance: if the two dimensions prove to be strongly correlated in representative samples, the second axis conveys little information beyond the first, and the model should be reduced to a single dimension (Table 3).

### 5.2 Four quadrants: diverse configurations of behavior change

By combining these two axes, behavior change can be described in terms of four quadrants (Figure 1; Table 4). These quadrants describe configurations of person, intervention, and context at a given point in time, and they are not categories of persons. The names are analytical shorthand; they are not proposed as labels to be applied to individuals, and they carry no judgment concerning the person who occupies the position described. Both axes are continuous, and the NOF proposes no cutoffs by which they might be divided into high and low. We therefore do not recommend median splits for research use. Where the two dimensions are used analytically, they should be entered as continuous variables together with their interaction, and where empirical typologies are sought, these should be derived from the data and not imposed upon it. The quadrants are also not fixed, since individuals may move between them over time, and supporting such movement is the practical purpose of the model.

**Table 4:** Definitions and hypothesized implications of the four quadrants.

Quadrant	Definition	Hypothesized implications and risks (to be tested)
Integrated success	A state in which behavioral outcomes improve and change, including lapses and fluctuations, is framed as part of an ongoing process, repaired within one’s narrative as needed, and held with a sense of authorship as part of the individual’s own story.	We predict that this configuration is the most durable of the four. Because change is held as the person’s own account, lapses are expected to remain repairable and behavior is expected to be more likely to be maintained after the intervention ends. This prediction has not been tested.
Brittle success	A state in which behavioral outcomes are favorable but the change is not framed as part of an ongoing process, not repaired within one’s narrative, and not held with authorship.	We predict that interruptions in this configuration are more likely to be fixed as self-blame, and that the risks of abrupt disengagement and of strain in the therapeutic relationship are elevated. Brittle describes the predicted stability of the configuration and not a property of the person.
Meaningful preparation	A state in which behavioral outcomes remain unachieved, yet lapses and stagnation are framed as part of an ongoing process and narratively repaired through trial and error, forming a basis for understanding change within one’s own life context.	We predict that re-engagement intentions and the therapeutic alliance are better preserved here than in the state below, and that this configuration is more likely to precede later behavioral change. Single-axis evaluation cannot distinguish this configuration from the one below.
Disempowered stagnation	A state in which behavioral outcomes remain unimproved and the components of narrative ownership are absent. The label does not denote a deficit, trait, or moral property of the person. Its source should be examined using the structural check, because low ownership may reflect constrained options, interpretive processes, or both.	We predict that this configuration more often reflects a mismatch between the intervention and the person’s circumstances than a limitation in interpretive capacity. Before any interpretive account is offered, the structural check described in Section 5.3 should be applied. Where options are materially constrained, this configuration should be read as an indicator of intervention or system failure.

These four quadrants describe configurations of person, intervention, and context at a given point in time. They are not categories of persons, diagnostic classifications, or evaluative judgments about individuals. Both dimensions are continuous, and the quadrants are illustrative conceptual configurations rather than groups defined by cutoffs (Section 5.2).

#### 5.2.1 Integrated success

Behavioral outcomes improve, and change is framed as part of an ongoing process, repaired when disruptions occur, and owned as part of the individual’s narrative. The NOF predicts that, because change is held as part of an individual’s own story, lapses remain repairable and the trajectory of change is more resilient and more likely to be sustained. This prediction has not been tested.

#### 5.2.2 Brittle success

Behavioral outcomes are favorable. However, this change is not owned by the individual. This is because the experience of change is not framed as a process, repaired, or owned as part of the individual’s narrative; this configuration is hypothesized to be more vulnerable to collapse following lapses or the withdrawal of external support.

#### 5.2.3 Meaningful preparation

Behavioral outcomes remain unachieved. However, lapses and stagnation are framed as part of an ongoing process and repaired through trial and error. This quadrant represents a stage in which conditions for future change are being formed, an aspect that cannot be captured within a single-axis evaluation that treats outcome non-achievement uniformly as failure.

#### 5.2.4 Disempowered stagnation

Both behavioral outcomes and core elements of narrative ownership remain absent. This suggests the need for a redesign of the intervention and renewed attention to individuals’ capacity to make sense of change.

### 5.3 Structural constraint and the individualization of disadvantage

A framework that locates a criterion of success in the way individuals interpret their own change carries a specific danger, namely that constraints belonging to circumstances are read as deficiencies belonging to persons. Health behavior is also shaped by structural conditions: income, housing, working conditions, caring responsibilities, discrimination, access to services, and food environments (56). An individual whose options are materially restricted may score low on narrative ownership for reasons that have nothing to do with interpretive capacity. Frameworks that overlook this become mechanisms of responsibilization, attributing to individuals what belongs to the conditions in which those individuals live (57, 58).

We therefore distinguish two sources of low narrative ownership within the NOF. Constrained low ownership arises where the option set itself is restricted, so that little remains over which authorship might be exercised. Interpretive low ownership arises where options are available but the experience of change has not been framed as a process, repaired within one’s narrative, or held with authorship (process framing, narrative repair, and authorship, respectively). The two are indistinguishable on the measure and can be distinguished only by examining the individual’s circumstances.

This distinction imposes a procedural requirement, which we term the structural check (Table 4). Before any interpretive account of low narrative ownership is offered, the opportunity structure available to the individual should be assessed, and indicators of material and temporal constraint should be measured alongside the construct in any study that uses it and reported together with it. Where constraint is present, low narrative ownership should be read as an indicator of the fit between the intervention and the individual’s circumstances, and therefore of intervention or system failure, and not as a characteristic of the individual.

When disempowered stagnation arises under constrained options, it is defined by what has been withheld from the individual—options, resources, and supportive conditions—and not by what the individual lacks. Where options are constrained, the appropriate response is to redistribute the burden of change through support resources, environmental modification, and relationships, before attention is given to the way in which the individual interprets a situation that was not of their choosing.

### 5.4 Evaluative implications and scope of application

The two-axis model extends the conventional evaluation by identifying previously invisible states and highlighting strategies that facilitate movement across quadrants. Supporting transitions from brittle success to integrated success, from disempowered stagnation to meaningful preparation, and from meaningful preparation to integrated success is therefore a central task of person-centered practice. The model identifies situations in which outcomes are favorable but change is not owned (brittle success). Those in which outcomes remain unachieved but narrative ownership persists (meaningful preparation) should be interpreted as progress rather than failure.

This interpretation is supported by theoretical traditions that recognize preparatory states as clinically meaningful. For example, the concept of recovery capital in addiction research (59, 60) and the theoretical foundations of Motivational Interviewing, which treat readiness for change as a form of clinical progress (61, 62), both acknowledge the significance of the preparatory phases preceding behavioral outcomes. The meaningful preparation quadrant provides a conceptual space for incorporating such insights into the evaluation.

For public health education and promotion, this model generates a practical theoretical implication: programs may succeed behaviorally while remaining fragile in terms of ownership, or may appear unsuccessful behaviorally while still building the interpretive conditions for later change. The NOF therefore provides a hypothesis-generating framework for strengthening program evaluation in settings where communication, education, digital support, and sustained engagement are central to public health impact.

Finally, the scope of application should be noted. The proposed model is particularly useful for behavioral domains that unfold over time and those that involve lapses or relapses, such as the self-management of chronic illness, smoking cessation, alcohol abstinence, and addiction recovery. In contrast, its application to one-time or acute behavior changes may require conceptual reconsideration. Furthermore, how the components of narrative ownership are expressed across different cultural contexts remains an important question for future research that aims to validate this model.

## 6 Using two criteria in program evaluation

If evaluation is to proceed along two dimensions, the manner in which those dimensions combine must be specified. The two dimensions should be reported separately and should not be merged into a weighted composite index, since a composite would conceal precisely the discrepancies that the NOF is designed to expose and would require a fixed exchange rate between the dimensions that no evidence currently supports. Table 5 sets out the resulting decision rules.

Where behavioral outcomes improve but narrative ownership is low, the program is not judged to have failed; the result is recorded as a conditional success, the intervention is retained, reflective components (opportunities for framing, repair, and articulation) are added, and the trajectory is monitored after external support has been withdrawn. Low narrative ownership is thus a reason to strengthen a program and never a reason to withhold an effective one.

The two dimensions are not equally weighted, and their relative priority is a property of the decision context and not of the framework itself. Where the behavior is directly and immediately consequential for health, outcome criteria retain priority and clinical escalation proceeds regardless of the level of narrative ownership. Where the question at issue is whether change will persist beyond the end of the program, the narrative ownership dimension carries greater weight. Evaluation protocols should therefore state this priority in advance, rather than resolving it once results are known.

Finally, narrative ownership does not offset harm. Although an individual may hold a health-damaging trajectory as fully their own, nothing in the NOF converts such a trajectory into a form of success; where outcomes deteriorate or the trajectory is harmful, harm-based criteria govern and ownership-focused support is adjunctive. This is stated as an absolute boundary of the NOF, because it is the point at which the framework would be most damaging if it were misapplied.

## 7 Discussion

### 7.1 Contribution in relation to existing work

The argument developed here converges with several existing lines of criticism, while differing from each of them in what it proposes. Critiques of behavior change research have long observed that measurable improvement is a partial account of what interventions accomplish (11), and existential and experiential approaches within human–computer interaction have argued that behavior change technologies neglect lived experience (21). These critiques are largely diagnostic. The NOF converts the diagnosis into a construct that carries a measurement model and criteria for disconfirmation, on the view that a criticism of evaluation which cannot itself be evaluated is unlikely to change the practice of evaluation.

The NOF also differs from theories of behavior change maintenance. Reviews of that literature identify motivational, self-regulatory, resource-related, habitual, and environmental mechanisms as determinants of whether behavior persists (9). Such accounts ask what sustains behavior. The present account asks what evaluation should count, and it proposes that a criterion currently absent from evaluation is itself among the determinants of whether behavior persists. The two are complementary; if the hypotheses stated above are supported, narrative ownership is at once a criterion of success and a mechanism of maintenance.

The NOF further stands in a specific relation to person-centered care and shared decision-making (15, 16). These traditions establish normative commitments concerning the manner in which care should proceed, but they supply no criterion by which programs may be judged to have honored those commitments. The two-axis model is offered as one such criterion, and its scope is deliberately narrow.

### 7.2 Limitations

Several limitations should be acknowledged. First, the NOF has not been empirically validated; no instrument measuring narrative ownership as defined here currently exists, and the predictions set out in Section 3.5 remain untested. What is offered is a research program and not a finding.

Second, conceptual overlap with adjacent constructs remains possible. The preceding sections have specified how narrative ownership differs from identity-based motivation, self-compassion, self-concordance, and related constructs; however, conceptual specification does not establish empirical separability, and the requirement of incremental validity set out in Table 2 may not be met.

**Table 2:** Proposed measurement model for narrative ownership and the validity evidence required.

Requirement	Proposed specification	Supporting evidence	Disconfirming evidence
Measurement structure	Three first-order factors (process framing, narrative repair, authorship), each measured reflectively; narrative ownership modeled as a second-order formative composite of the three, because the components are not interchangeable indicators and removing one alters the meaning of the construct (45)	Acceptable fit for each first-order reflective model; adequate weights for all three components in the composite	One or more components carry a non-significant weight in the composite across samples, indicating that the component is not constitutive
Relations among components	Moderate positive intercorrelations, in the range conventionally described as medium to large (52), and clearly below the level at which discriminant validity between components would be questioned	Correlations in the predicted range with distinct factor loadings for each component	Heterotrait–monotrait ratios among the three components at or above the conservative benchmark of 0.85 (51), indicating that the components are not empirically separable and that a single reflective factor should be preferred
Scoring	Report the three subscale scores as a profile. A single averaged total is not recommended, because the components are formative and equal weighting is not assumed	Predictive models using the three components outperform a unit-weighted total	A unit-weighted total performs equivalently, in which case the simpler score should be preferred
Convergent validity	Moderate associations with narrative identity self-evaluation, self-compassion, and meaning-making measures (40, 42, 49)	Correlations of moderate magnitude in the expected direction	Near-zero associations, indicating that the construct is not measuring what it claims
Discriminant validity	Associations with self-efficacy and autonomous motivation lower than the associations among the three components	Heterotrait–monotrait ratios below the conservative benchmark of 0.85 (51) in independent samples	Ratios at or above 0.85, or at or above the level of the components’ own intercorrelations
Criterion validity	Prospective association with post-lapse re-engagement and 12-month maintenance	Association retained after adjustment for baseline behavior and end-of-intervention outcome level	No association after adjustment
Incremental validity	Prediction of post-lapse re-engagement over and above self-efficacy, autonomous motivation, self-concordance, self-compassion, and psychological flexibility	Significant increment in explained variance in pre-registered models	No increment, in which case narrative ownership should be regarded as redundant with existing constructs
Longitudinal measurement	Configural, metric, and scalar invariance across at least three measurement occasions (47)	Invariance supported at the scalar level	Failure of metric invariance, which would preclude interpreting change scores
Sensitivity to change	Responsiveness to reflective intervention components with a detectable manipulation effect	Between-arm difference on subscale scores in a randomized design	No difference despite a successful reflective manipulation
Cross-cultural applicability	Invariance testing across at least one individualist and one collectivist context, given that the expression of authorship may be culturally patterned (63)	Partial invariance sufficient for comparison	Substantive non-invariance, in which case the construct requires culture-specific specification

The numerical benchmarks cited here are conventions drawn from the measurement literature rather than properties of the construct, and are stated in advance so that the measurement claims of the framework can be adjudicated rather than asserted.

**Table 3:** Operational specification of the three hypotheses.

Specification	H1 (predictive)	H2 (intervention responsiveness)	H3 (experiential and relational consequences)
Statement	Among individuals with equivalent behavioral outcomes at the end of an intervention, higher narrative ownership predicts greater 12-month maintenance and a higher probability of post-lapse re-engagement	Adding reflective support components to a digital behavior change intervention improves medium- to long-term maintenance, and this effect is mediated by narrative ownership rather than by engagement in general	Among individuals who have experienced a lapse, higher narrative ownership is associated with lower self-stigma and better-preserved therapeutic alliance, with the association stronger in stigmatized or relapse-prone behavioral domains
Design	Prospective cohort following a completed structured behavior change program	Randomized controlled trial with an engagement-matched active control arm	Longitudinal observational study with repeated measurement following a lapse
Population	Adults completing a program targeting a behavior that unfolds over time (chronic disease self-management, smoking cessation, alcohol reduction)	Users of a digital behavior change intervention in the same behavioral domains	Individuals who have experienced at least one lapse during an ongoing behavior change attempt
Predictor or manipulation	Narrative ownership subscale scores measured at end of intervention	Reflective prompts inviting the user to situate lapses within an ongoing account, versus an active control matched on contact frequency, time on task, and message volume but containing no reflective content	Narrative ownership subscale scores measured within 14 days of lapse onset
Operational definitions	Lapse: for abstinence-based goals, a single episode of the proscribed behavior, following the standard lapse–relapse distinction in relapse prevention research (5, 13, 27). For behaviors requiring repeated performance, we propose an analogous criterion of seven or more consecutive days without the target behavior. The seven-day interval is adapted from the convention used to define relapse in smoking cessation trials (64), where it denotes seven consecutive days of the proscribed behavior rather than its absence; the adaptation is offered as a worked example and requires domain-specific justification. Post-lapse re-engagement: resumption of the target behavior meeting the pre-specified behavioral criterion and sustained for a pre-specified minimum duration, within a pre-specified window following lapse onset; the length of this window should be derived from the time course of the behavior in question rather than set by convention. Maintenance: meeting the pre-specified behavioral criterion at 12 months after the intervention ends	As for H1, with the addition of a manipulation check on the three subscales at weeks 4 and 8	Self-stigma measured with a validated domain-appropriate scale; therapeutic alliance measured with a validated working-alliance measure
Timing	Measurement at end of intervention and at 3, 6, and 12 months	Measurement at baseline, weeks 4 and 8, and at 6 and 12 months	Measurement within 14 days of lapse and at 3 and 6 months
Covariates	Baseline behavior, end-of-intervention outcome level, self-efficacy, autonomous motivation, self-compassion, psychological flexibility, and indicators of structural constraint (income adequacy, work schedule control, care responsibilities, access to services)	As for H1, with baseline outcome and platform engagement	Behavioral domain, lapse severity, prior lapse history, self-compassion, and the same indicators of structural constraint
Analysis	Multilevel or discrete-time survival models for maintenance and re-engagement, with pre-registered adjustment sets	Mediation analysis with narrative ownership and platform engagement entered as competing mediators, using bootstrapped indirect effects	Longitudinal models testing the main association and the interaction with behavioral domain
Result that would disconfirm	Narrative ownership shows no association with maintenance or re-engagement after adjustment, or the association is fully accounted for by self-efficacy and autonomous motivation	The reflective arm improves outcomes without altering narrative ownership scores, or the indirect effect operates through engagement while the indirect effect through narrative ownership does not differ from zero	No association in stigmatized or relapse-prone domains, or the association is fully accounted for by self-compassion

The framework as a whole is disconfirmed if narrative ownership fails the incremental validity requirement in Table 2, or if the results specified in the final row are obtained across pre-registered replications. As a further falsification criterion for the two-axis structure, we specify in advance that a correlation of approximately.70 or above between the two dimensions in representative samples would indicate that the second axis conveys little information beyond the first, and that the framework should be reduced to a single dimension. This criterion is adopted as a deliberately stringent heuristic for redundancy rather than as an established discriminant-validity cutoff. The operational criteria in this table are proposed as worked examples to render the hypotheses testable in principle. They are not presented as established standards, and each should be justified and pre-registered with reference to the behavior under study.

**Table 5:** Dual-criterion decision rules for program evaluation.

Observed pattern	Evaluative interpretation	Recommended response	Boundary condition
Behavioral outcomes improve; narrative ownership high or increasing	Integrated success	Report both dimensions; sustain and taper external support gradually	—
Behavioral outcomes improve; narrative ownership low or decreasing	Conditional success requiring ownership support. The program is not judged to have failed, but the result should not be recorded as unqualified success	Retain the program and add reflective and meaning-linking components; monitor after support is withdrawn	This is not a reason to withhold an effective intervention. Behavioral benefit is not discounted because ownership is low
Behavioral outcomes unachieved; narrative ownership high or increasing	Progress that single-axis evaluation records as failure	Continue; re-examine goal fit, timing, and barriers rather than discontinuing on outcome grounds alone	Where the behavior is time-critical for health, for example medication adherence in acute or high-risk conditions, outcome criteria retain priority and clinical escalation is required regardless of ownership
Behavioral outcomes unachieved; narrative ownership low	Apply the structural check before any interpretive account is offered. Where options are materially constrained, the finding indexes intervention or system failure	Redesign the distribution of burden (support resources, environment, and relationships) before addressing interpretive processes	Attributing this pattern to the person’s interpretive processes without first assessing the opportunity structure individualizes structural disadvantage
Behavioral outcomes deteriorate, or the trajectory is health-damaging; narrative ownership high	Failure on the outcome dimension. Narrative ownership does not offset harm	Address the behavioral risk directly; ownership-focused support is adjunctive and not substitutive	Narrative ownership is never a substitute for health outcomes. A person can hold a harmful trajectory as their own account. Harm-based criteria override the ownership dimension in all cases

The two dimensions should be reported separately and should not be combined into a weighted composite index. The relative priority of the two dimensions is a function of the decision context rather than a fixed property of the framework, and should be specified in advance in the evaluation protocol. The structural check refers to the assessment of the opportunity structure described in Section 5.3.

Third, measurement invariance is uncertain. Whether the construct can be measured equivalently across behavioral domains, across time, and across populations is unknown, and a failure of invariance would restrict the use of the NOF to within-group comparison.

Fourth, authorship may be culturally patterned. The NOF was developed by authors working in Japan, where the self is more commonly articulated in relational than in individual terms (63), and the expression of change as one’s own story may not be directly comparable across cultural contexts. Whether authorship is a culturally general property, or a culturally specific idiom of a more general phenomenon, is an empirical question that cannot be settled here.

Fifth, the risk of individualizing structural disadvantage is real and is not eliminated by the safeguards proposed in Section 5.3. The structural check is a procedural requirement, and procedural requirements can be disregarded by those who find the individual-level reading of the NOF more convenient.

Sixth, the translation of two continuous dimensions into four named quadrants invites categorical use, notwithstanding the cautions stated in Section 5.2. Names of this kind are useful for communication and are liable to be treated as classifications.

Seventh, the scope of the NOF is bounded. The NOF addresses behaviors that unfold over time and that admit of lapse, and its application to single-occasion or acute behaviors, and to behaviors performed under conditions of acute clinical urgency, requires reconsideration and not extension.

### 7.3 Future directions

The immediate priority is instrument development, following the requirements set out in Table 2, with initial validation in a domain in which lapses are frequent and consequential. Two further steps follow: a longitudinal cohort study testing whether narrative ownership predicts maintenance and post-lapse re-engagement beyond established predictors, and a randomized controlled trial with an engagement-matched control condition testing whether reflective components alter the construct specifically. Qualitative research is also needed, both to establish that the three components are recognizable in the way people actually speak about change and to examine whether the construct behaves as described among individuals whose options are materially constrained.

## 8 Conclusion

This study reconsidered outcome-centered success evaluation in behavior change research in light of the normative commitment of person-centered care and shared decision-making. As technologies for influencing behavior become increasingly sophisticated, the key question is no longer whether behavior can change. Rather, the more fundamental question is ethical and interpretive: whether such change, when owned by the individual, can be re-narrated as part of an experience that includes lapses and can be taken up as part of one’s life. Evaluation structures that treat outcomes alone as successes embody the three layers of ethical fragility analyzed in Section 4.

The NOF provides a conceptual evaluation framework that incorporates narrative ownership as a second dimension of success, while retaining the empirical achievements represented by behavioral outcomes. The proposed two-axis model reframes behavior change not merely as a numerical improvement, but as a process whose resilience can be empirically tested, in which lapses are framed as part of lived experience and repaired through the individual’s narrative. Therefore, the NOF offers a starting point for rethinking success in terms of effectiveness and ownership, and for empirically testing whether sustainable health behavior change depends not only on what people do, but also on whether change is narratively held as their own.

## 9 Practice implications

First, to detect brittle success at an early stage and facilitate movement toward integrated success, public health educators, clinicians, and program evaluators should not rely solely on favorable objective indicators, such as laboratory values or physical activity levels. Even when outcomes appear favorable, it is necessary to examine whether the change aligns with the individual’s values and is experienced as owned, framed, and articulated as part of the individual’s own narrative (15). For example, healthcare professionals may ask how improvements in the clinical indicators relate to the patient’s priorities (e.g., “what really matters in life”), thereby helping patients frame and articulate behavior changes within their personal narratives.

Second, to support the movement from disempowered stagnation to meaningful preparation, lapses and interruptions should be framed not as simple failures, but as opportunities for narrative reconstruction and learning (27, 54). Healthcare professionals can play a key role in helping individuals reflect on how lapses are experienced and reinterpreted within the broader context of everyday life. Questions that encourage reflection on what has been learned from the lapse can help shift the narrative from self-blame to learning and reengagement.

Third, to support the movement from meaningful preparation to integrated success, the design of digital health applications should incorporate opportunities for users to connect behavioral goals with personal meanings that motivate them (44). For example, applications could display user-defined meanings alongside behavioral goals, linking daily actions to the user’s broader life narratives to enhance the overall experience and relevance.

## Data Availability

The original contributions presented in the study are included in the article/supplementary material, further inquiries can be directed to the corresponding author.
